# miRNome of inflammatory breast cancer

**DOI:** 10.1186/1756-0500-7-871

**Published:** 2014-12-04

**Authors:** Diana V Maltseva, Vladimir V Galatenko, Timur R Samatov, Svetlana O Zhikrivetskaya, Nadezhda A Khaustova, Ilya N Nechaev, Maxim U Shkurnikov, Alexey E Lebedev, Irina A Mityakina, Andrey D Kaprin, Udo Schumacher, Alexander G Tonevitsky

**Affiliations:** SRC Bioclinicum, Ugreshskaya str 2/85, 115088 Moscow, Russia; Moscow State University, Leninskie Gory, 119991 Moscow, Russia; P.A. Hertsen Moscow Research Oncology Institute, 2nd Botkinskii p. 3, Moscow, 125284 Russia; Department of Anatomy and Experimental Morphology, University Cancer Center, University Medical Center Hamburg-Eppendorf, Martinistr. 52, Hamburg, D-20246 Germany

**Keywords:** Inflammatory breast cancer, miRNA, Microarray, tp53 mutational status

## Abstract

**Background:**

Inflammatory breast cancer (IBC) is an extremely malignant form of breast cancer which can be easily misdiagnosed. Conclusive prognostic IBC molecular biomarkers which are also providing the perspectives for targeted therapy are lacking so far. The aim of this study was to reveal the IBC-specific miRNA expression profile and to evaluate its association with clinicopathological parameters.

**Methods:**

miRNA expression profiles of 13 IBC and 17 non-IBC patients were characterized using comprehensive Affymetrix GeneChip miRNA 3.0 microarray platform. Bioinformatic analysis was used to reveal IBC-specific miRNAs, deregulated pathways and potential miRNA targets.

**Results:**

31 differentially expressed miRNAs characterize IBC and mRNAs regulated by them and their associated pathways can functionally be attributed to IBC progression. In addition, a minimal predictive set of 4 miRNAs characteristic for the IBC phenotype and associated with the TP53 mutational status in breast cancer patients was identified.

**Conclusions:**

We have characterized the complete miRNome of inflammatory breast cancer and found differentially expressed miRNAs which reliably classify the patients to IBC and non-IBC groups. We found that the mRNAs and pathways likely regulated by these miRNAs are highly relevant to cancer progression. Furthermore a minimal IBC-related predictive set of 4 miRNAs associated with the TP53 mutational status and survival for breast cancer patients was identified.

**Electronic supplementary material:**

The online version of this article (doi:10.1186/1756-0500-7-871) contains supplementary material, which is available to authorized users.

## Background

Inflammatory breast cancer (IBC) is an extremely malignant form of breast cancer characterized by early metastases formation and high lethality with the 10 year-survival not exceeding 30% [1]. For one third of patients distant metastases are already detected within 3 months after first symptoms appeared [2]. As IBC does not produce solid tumors it can easily be misdiagnosed as mastitis or bacterial infection [3]. This makes independent IBC molecular markers having predictive and prognostic value and providing the perspectives for targeted therapy highly desirable.

Today a number of studies on molecular characterization of IBC patient samples have been published including several genome-wide transcriptomic analyses [4–9]. However, the suggested mRNA biomarkers differed from one study to another possibly indicating the heterogeneous nature of IBC.

MiRNAs are small non-coding RNAs regulating gene expression which are involved in diverse biological processes [10]. MiRNAs proved to be reliable markers of various diseases including cancers [11]. Recently two pioneering studies have been published discovering miRNA profile of IBC using PCR-based approach [12, 13]. Although they have analyzed a limited number of miRNAs and identified completely different profiles, they provided initial insight in the miRNAs regulating RNA networks characteristic for IBC and highlighted the potential of miRNAs as IBC molecular biomarkers.

The aim of the present study was to reveal the complete miRNome and regulated miRNA-mRNA networks of the IBC. Samples from IBC and non-IBC patients analyzed by the Affymetrix GeneChip miRNA 3.0 microarray platform formed the basis for this study. Differentially expressed miRNAs and pathways regulated by them were revealed. In addition a minimal predictive set of miRNAs characteristic for IBC phenotype was identified taking clinicopathological parameters of breast cancer patients into consideration.

## Methods

### Ethics statement

The study was approved by the ethics committee of Scientific Research Center Bioclinicum (Moscow, Russia) and all participants signed an informed consent statement.

### Patients and material

Tumor samples were collected from 30 women comprising 13 IBC and 17 non-IBC patients. IBC was diagnosed according to the well-accepted criteria described in the AJCC Cancer Staging Manual [14]. IBC patients presented with diffuse enlargement of the involved breast as well as erythema and oedema of the skin above it. Immediately after surgery the tumor samples were stored in RNAlater buffer (Qiagen, Germany) at -80°C until RNA extraction.

Tumor samples characteristics are presented in Table 1. One patient was assigned to each of IIB, III, IIIC and IV stage subgroups and nine patients were staged as IIIB subgroup within the IBC group whereas non-IBC patients tended to have moderate stages (4 stage I, 1 II, 8 IIA, 1 IIB, 2 IIIA and 1 IIIC). Remarkably 15% of IBC patients (2 out of 13) had already distant metastases while no metastases were detected among non-IBC group. These data are consistent with the known IBC aggressiveness.

**Table 1:** **Clinico-pathological characteristics of breast cancer samples**

	Breast carcinomas		p-value ^a,b^
	nonIBC	IBC	
Characteristics (nb,%)	(n = 17)	(n = 13)	
**Age**			
Mean ± SD	57.4 ± 12.4	54.4 ± 13.4	0.27^a^
≤50	5 (29%)	5 (38%)	0.71^b^
>50	12 (71%)	8 (62%)	
**Stage**			
I	4 (24%)	0 (0%)	<0.001^b^
II	1 (6%)	0 (0%)	
IIA	8 (47%)	0 (0%)	
IIB	1 (6%)	1 (8%)	
III	0 (0%)	1 (8%)	
IIIA	2 (12%)	0 (0%)	
IIIB	0 (0%)	9 (69%)	
IIIC	1 (6%)	1 (8%)	
IV	0 (0%)	1 (8%)	
**Distant metastates**			
Yes	0 (0%)	2 (15%)	0.18^b^
No	17 (100%)	11 (85%)	
**Estrogen receptor status**			
Positive	12 (71%)	5 (38%)	0.14^b^
Negative	5 (29%)	8 (62%)	
**Progesterone receptor status**			
Positive	9 (53%)	2 (15%)	0.06^b^
Negative	8 (47%)	11 (85%)	
**HER2 status**			
Positive	4 (24%)	2 (15%)	0.67^b^
Negative	13 (76%)	11 (85%)	
**Molecular subtypes**			
HR^-^HER2^-^	3 (18%)	7 (54%)	0.19^b^
HR^-^HER2^+^	1 (6%)	1 (8%)	
HR^+^HER2^-^	10 (59%)	4 (31%)	
HR^+^HER2^+^	3 (18%)	1 (8%)	

^a^Student’t test, ^b^Fisher’s exact test.

HR: Hormone Receptor; HR^-^: ER and PR negative; HR^+^: ER and/or PR positive.

### RNA extraction

Total RNA was extracted from the breast tissue using miRNeasy Mini Kit (Qiagen, Germany) as recommended by the manufacturer. RNA concentrations were determined by the Nanodrop photometer (NanoDrop, USA). RNA quality was checked using the Agilent Bioanalyser 2100 System (Agilent Technologies, USA). For all samples RNA integrity number (RIN) was greater than 7.

### Microarray analysis

For complete miRNome profiling the samples were prepared using FlashTag Biotin HSR RNA Labeling Kit as recommended by the manufacturer [15]. The samples were hybridized on GeneChip miRNA 3.0 Arrays (Affymetrix) for 16 h at 48°C. Arrays were washed to remove non-specifically bound nucleic acids and stained on Fluidics Station 450 (Affymetrix) and then scanned on GeneChip Scanner 3000 7G system (Affymetrix).

### Microarray data processing and bioinformatic analysis

GeneChip miRNA 3.0 microarrays were processed using Affymetrix Expression Console (version 1.3.1) [15] implementation of Robust Multichip Average (RMA) method [16]. The processing included background adjustment based on a global model for the distribution of probe intensities [16], quantile normalization [17] and summarization based on Tukey’s median polish procedure [18].

The detection of differentially expressed transcripts was restricted to human miRNAs, the signals from all other microarray probesets were ignored. The detection of differentially expressed miRNAs was performed using Bioconductor [19] package limma [20]: moderated t-test [21] was applied to log-scaled expression values, and the thresholds were set to 0.01 for the p-value and 1.2 for the fold change.

**Figure 1:**
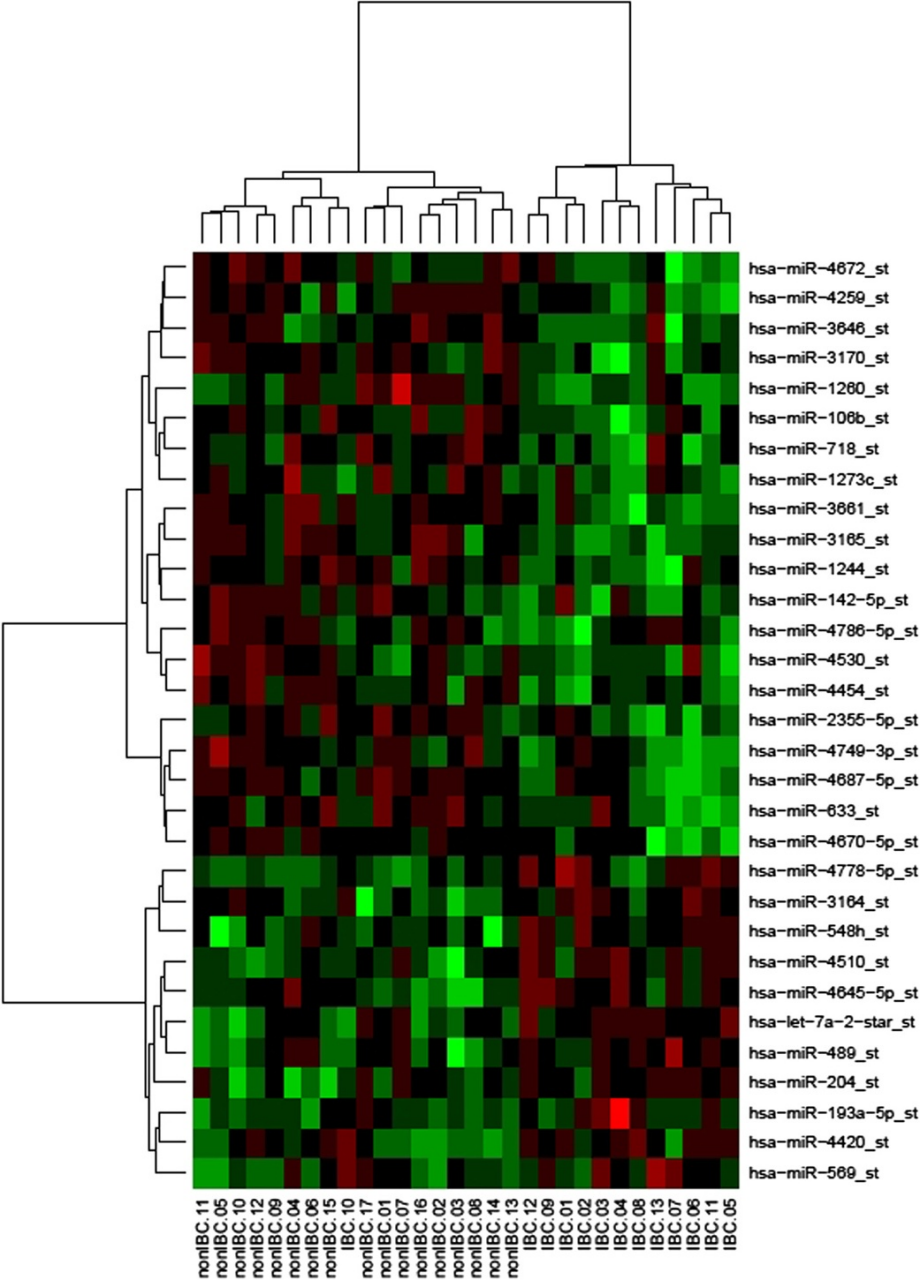
**Cluster analysis heatmap for 13 IBC and 17 non-IBC samples based on the expression profile of the 31 differentially expressed miRNAs.** The expression data are represented in a 2D format, with rows indicating miRNAs and columns indicating samples. High expression values are coded with red color and low expression values are coded with green.

Hierarchical clusterization for the heatmap (Figure 1) was constructed based on the normalized log-scaled expression values (i.e., log-scaled expression values decreased by the mean value and divided by standard deviation) using Euclidean distance and average cluster method. The construction was performed by the Heatmap online service [22] that utilizes the heatmap tool of R package gplots [23]. For the hierarchical sample clusterization the p-value indicating the association of two resulting clusters with IBC status was obtained using one-sided binomial test. This p-value is the probability of observing the same or better classification accuracy for a classifier with no information rate, i.e., a classifier that attributes a sample to a class with the probability equal to the class percentage in the data.

The validated target mRNAs of differentially expressed miRNAs were found using TARBASE [24], miRecords [25] and miRTarBase [26] databases. The identification of the enriched pathways was performed using DAVID online service [27, 28].

The construction of a set of 4 miRNAs for the classification of IBC vs. non-IBC samples was performed using the approach of Galatenko et al. [29]. This approach is based on the Support Vector Machine [30] with linear kernel and greedy-type transcript selection. During the construction the set of the samples was randomly divided into a training set and a testing set. MiRNA log-scaled expressions were normalized using mean expression and standard deviation that were calculated based on the training set. The classifier construction utilized R packages Kernlab [31] and Caret [32].

The assessment of the connection between resulting classifier values and TP53 mutational status for the GSE19536 dataset [33] was performed as follows. The expression values of 4 selected miRNAs were normalized using mean expression and standard deviation that were calculated based on the GSE19536 expression matrix downloaded from the Gene Expression Omnibus data repository. MiRNAs with no expression values were considered to have zero expression and normalized expression was set to zero for these miRNAs. Then classifier values (or, more precisely, values of the linear combination utilized for the classification) were calculated using normalized expression values without any changes in classifier coefficients. Finally, the set of classifier values associated with TP53-mutated samples was compared with the set of classifier values associated with TP53-wild type samples using Mann–Whitney U-test.

## Results and discussion

### MiRNA differential expression profile

The IBC-specific profile of miRNA expression has been identified using comprehensive Affymetrix GeneChip miRNA 3.0 microarray platform. The expression data for 1733 miRNAs are presented in the Additional file 1. 31 miRNAs with the fold change of at least 1.2 times and the p-value not higher than 1% between non-IBC and IBC groups were considered to be differentially expressed and are listed in the Table 2.

**Table 2:** **Differentially expressed miRNAs**

miRNA	Fold change value	p-value	Adjusted p-value
**up-regulated in IBC**			
hsa-miR-3165	1.32	2.4 × 10^-5^	0.04
**hsa-miR-4687-5p**	**1.62**	**1.1 × 10** ^**-4**^	**0.06**
**hsa-miR-4259**	**1.52**	**1.4 × 10** ^**-4**^	**0.06**
hsa-miR-3661	1.41	4.6 × 10^-4^	0.15
**hsa-miR-4749-3p**	**1.57**	**5.1 × 10** ^**-4**^	**0.15**
hsa-miR-3170	1.31	9.2 × 10^-4^	0.22
**hsa-miR-4672**	**1.53**	**1.1 × 10** ^**-3**^	**0.22**
hsa-miR-633	1.27	1.2 × 10^-3^	0.22
**hsa-miR-4454**	**1.80**	**1.3 × 10** ^**-3**^	**0.22**
**hsa-miR-1260**	**1.67**	**1.9 × 10** ^**-3**^	**0.28**
hsa-miR-4670-5p	1.31	2.3 × 10^-3^	0.30
**hsa-miR-718**	**1.76**	**2.4 × 10** ^**-3**^	**0.30**
**hsa-miR-106b**	**1.56**	**2.9 × 10** ^**-3**^	**0.31**
**hsa-miR-1244**	**2.29**	**3.2 × 10** ^**-3**^	**0.31**
**hsa-miR-4530**	**1.53**	**5.5 × 10** ^**-3**^	**0.43**
**hsa-miR-4786-5p**	**2.35**	**5.9 × 10** ^**-3**^	**0.43**
hsa-miR-3646	1.49	6.1 × 10^-3^	0.43
hsa-miR-142-5p	1.24	7.9 × 10^-3^	0.47
hsa-miR-2355-5p	1.28	8.0 × 10^-3^	0.47
**hsa-miR-1273c**	**1.57**	**8.1 × 10** ^**-3**^	**0.47**
**down-regulated in IBC**			
**hsa-miR-4778-5p**	**1.72**	**7.0 × 10** ^**-5**^	**0.06**
hsa-miR-3164	1.39	1.8 × 10^-3^	0.28
hsa-miR-4645-5p	1.44	3.0 × 10^-3^	0.31
hsa-miR-4420	1.26	3.2 × 10^-3^	0.31
**hsa-miR-4510**	**1.63**	**3.5 × 10** ^**-3**^	**0.32**
hsa-miR-548h	1.24	6.1 × 10^-3^	0.43
hsa-miR-569	1.45	6.2 × 10^-3^	0.43
hsa-let-7a-2-star	1.33	7.2 × 10^-3^	0.47
hsa-miR-204	1.45	7.3 × 10^-3^	0.47
**hsa-miR-193a-5p**	**1.59**	**9.0 × 10** ^**-3**^	**0.50**
**hsa-miR-489**	**2.14**	**9.4 × 10** ^**-3**^	**0.51**

The miRNAs with p-value <0.01 and fold change >1.5 are marked with bold.

Remarkably, the majority of them are known to be associated with breast cancer, and the up-regulation of highly expressed miRNAs is linked with more aggressive phenotype and poor prognosis. More specifically, hsa-miR-3165, hsa-miR-4687-5p, hsa-miR-3661, hsa-miR-4749-3p, hsa-miR-3170, hsa-miR-4672, hsa-miR-4670-5p, hsa-miR-4786-5p, and hsa-miR-2355-5p have been detected in breast cancer when compared with normal breast tissue [34]. Circulating hsa-miR-718 was suggested to be a fluid biomarker for breast cancer [35]. Expression of hsa-miR-106b is elevated in higher stage tumors and correlated with tumor progression [36]. Hsa-miR-142-5p has been demonstrated to be up-regulated in lymph node breast cancer patients [37].

Other up-regulated miRNAs have been associated with different types of malignomas including hsa-miR-1260 and hsa-miR-1273c for melanoma [38], hsa-miR-633 for endometrial cancer [39], hsa-miR-1244 for hepatocellular carcinoma [40], hsa-miR-4454 and hsa-miR-4530 for malignant B cells [41].

The miRNAs down-regulated in IBC group include hsa-miR-204 which has been characterized as a tumor suppressor in breast cancer [42], ovarian cancers and pediatric renal tumors [43]. Hsa-miR-193a-5p is also less expressed in IBC patients and is known to play a suppressive role in breast cancer [44]. Another down-regulated miRNA is hsa-miR-489 targeting Smad3 transcription factor thus inhibiting epithelial-mesenchymal transition of breast cancer cells which prevents metastases formation and makes the cells more susceptible to chemotherapy [45]. The let-7 family of miRNAs is reduced in rare self-renewing breast tumor-initiating cells [46].

Consistent with the expression level of the above listed miRNAs, down-regulated hsa-miR-548h is more abundant in normal lung tissue than in lung cancer [47], and hsa-let-7a-2 is less expressed in aggressive hepatocellular carcinoma [48] and its down-regulation is correlated with poor survival in lung cancer [49].

The hierarchical clustering of breast cancer samples according to the differentially expressed miRNAs is presented in Figure 1. Remarkably, the patients were indeed separated into non-IBC and IBC clusters with the only one IBC sample misclassified resulting in the p-value of 9.5 × 10^-7^.

All these data are consistent with the more aggressive nature of IBC and suggest the differentially expressed miRNAs to be molecular biomarkers for IBC. Notably, the revealed miRNA pattern does not overlap with the previously identified profiles [12, 13]. This can be explained by the larger number of miRNAs covered by the comprehensive microarrays used in this study as compared to both previous studies.

During IBC progression breast tumors are infiltrated by inflammatory cells, in particular monocytes/macrophages [50, 51]. We checked miRNA profile of these cells which has been recently published [52]. Only 6 out of 20 up-regulated in IBC miRNAs are pronouncedly expressed in macrophages, namely hsa-miR-142-5p, hsa-miR-106b, hsa-miR-4454, hsa-miR-2355-5p, has-miR-1273c and hsa-miR-4687-5p (Table 2). This moderate intersection clearly indicates that the identified miRNA profile is indeed IBC-specific and cannot be due to the migrated macrophages.

### mRNA targets of differentially expressed miRNAs

The mRNAs which have been validated to be targets for the differentially expressed miRNAs were found using online databases as described in Methods. All 31 differentially expressed miRNAs have 428 target mRNAs in total. We performed pathway enrichment analysis for these genes (Table 3). The top of revealed pathways includes phosphoproteins, regulation of kinase activity and cell proliferation all known to be highly relevant to malignant progression. Besides, acetylation, transcription, DNA binding and protein biosynthesis are the key interrelated steps in gene expression and their deregulation is involved in cancer progression [53].

**Table 3:** **Selected pathways highly enriched with the validated target genes of differentially expressed miRNAs**

Pathway	Number of genes	Adjusted P-value
Phosphoprotein	256	2.3 × 10^-18^
Acetylation	127	8.0 × 10^-17^
Transcription	86	1.4 × 10^-6^
Regulation of kinase activity	28	4.6 × 10^-4^
DNA binding	100	4.6 × 10^-4^
Regulation of cell proliferation	45	6.9 × 10^-4^
Protein biosynthesis	17	2.3 × 10^-4^

The same mRNA can be targeted by more than one miRNA thus providing for more efficient and specific regulation [54]. Table 4 lists the mRNAs regulated by 2 differentially expressed miRNAs. These 11 mRNAs are more likely involved in the IBC progression.

**Table 4:** **mRNAs targeted with 2 differentially expressed miRNAs**

miRNAs	Validated target genes
hsa-miR-106b ↑	*APLP2, APP, CDKN1A, EEF1A1, PRMT3*
hsa-miR-1260 ↑	
hsa-miR-106b ↑	*ELOVL6, IPO7*
hsa-miR-204 ↓	
hsa-miR-1260 ↑	*ENO1, RPL3*
hsa-miR-204 ↓	
hsa-let-7a-2-star ↓	*HMGA2*
hsa-miR-204 ↓	
hsa-miR-106b ↑	*VEGFA*
hsa-miR-548 h ↓	

Up- and down-regulated miRNAs in IBC vs. non-IBC patients are indicated by the upward and downward arrows, respectively.

More specifically, amyloid beta precursor-like protein 2 (APLP2) is targeted by hsa-miR-106b and hsa-miR-1260. These miRNAs are up-regulated in IBC thus potentially suppressing the expression of this gene. Notably, hsa-miR-106b and hsa-miR-1260 follow the more stringent criteria in t-test, namely p-value <0.01 and fold change >1.5, supporting their functional relevance. Remarkably, APLP2 mRNA is known to be down-regulated in neuroendocrine tumors and lung cancer [55, 56]. The same two miRNAs target amyloid beta precursor protein (APP) which has been identified as a hub protein in differentially expressed networks between ER+ and ER- breast cancer patients [57]. Another target of these miRNAs is cyclin-dependent kinase inhibitor 1A (CDKN1A). Low expression of this gene in breast cancer patients is associated with poor survival after chemotherapy which is consistent with the aggressiveness of IBC [58]. Hsa-miR-106b and hsa-miR-1260 also regulate alpha 1 subunit of eukaryotic translation elongation factor 1 (EEF1A1). This protein is involved in regulation of epithelial-mesenchymal transition in breast cancer cells [59]. At the same time EEF1A1 has been identified as a reliable reference gene for quantitative PCR assay of breast cancer patient biopsies, i.e. this mRNA is equally abundant across multiple breast cancer samples [60]. Finally these two miRNAs target protein arginine methyltransferase 3 (PRMT3) mRNA, an enzyme interacting with DAL-1/4.1B protein thus inducing apoptosis in breast cancer cells [61].

Fatty acid elongase 6 (ELOVL6) is targeted by hsa-miR-106b and hsa-miR-204. The expression of these miRNAs is changed in opposite directions implying balanced and delicate regulation of the target mRNA. The functional link of this rate-limiting enzyme of *de novo* lipogenesis to the breast tumorigenesis has been revealed using mouse models [62]. The expression of the protein of nuclear import importin 7 (IPO7) is also regulated by these oppositely directed miRNAs. This protein is known to be involved in the regulation of prostate cancer cells proliferation [63]. Additionally the importin 7-mediated nuclear import plays an important role in the keratin 19 tumor suppressor mechanism in breast cancer cells [64].

The mRNA of enolase 1 (ENO1) is targeted by both up-regulated hsa-miR-1260 and down-regulated hsa-miR-204. The increased level of this protein is associated with a poor prognosis for breast cancer patients and involved in tamoxifen and methotrexate resistance of breast cancer cells [65, 66]. Another target of these miRNAs is the gene RPL3 coding for the ribosomal protein L3. This protein is a member of the Pes1-Bop1 complex involved in the colorectal tumorigenesis [67].

The decreased in IBC patients hsa-let-7a-2-star and hsa-miR-204 are expected to increase the level of the transcriptional regulator high mobility group AT-hook protein 2 encoded by the *HMGA2* gene. Remarkably, the rare self-renewing breast tumor-initiating cells have been described to contain more of HMGA2 mRNA in combination with reducing of its regulator hsa-let-7a-2-star [46]. Also recently the overexpression of this gene was found to make the breast cancer cells more metastatic [68, 69].

Finally the VEGFA mRNA is targeted by the oppositely directed hsa-miR-106b and hsa-miR-548 h. This gene encodes vascular endothelial growth factor A, a protein with well-established role in cancer progression. Its increased expression is associated with loss of wild type tp53 status and predicts poor outcome for the breast cancer patients [70] which can functionally be explained by the induction of angio- and lymphangiogenesis [71]. Recently an angiogenesis-independent function of VEGFA has been reported, namely the protein produced by tumor cells can act in an autocrine manner to promote cell growth, and reducing its expression resulted in a differentiated phenotype *in vitro* and inhibited tumor forming capacity *in vivo* [72].

The presented data support the functional relevance of the revealed miRNA-mRNA networks to the IBC progression and denote potential targets for the IBC-specific therapy.

### A predictive set of 4 miRNAs associated with TP53 mutation status

As IBC is characterized by high aggressiveness and poor survival we tried to identify a minimal set of miRNAs which could reliably classify patients to IBC or non-IBC group and have overall predictive value for breast cancer patients. We have investigated sets of 4 miRNAs using a bioinformatic approach based on the Support Vector Machine [30] with linear kernel and greedy-type transcript selection as described in Methods. The collection of the samples was randomly divided into a training set and a testing set. The classifier was constructed based solely on the training set which contained 7 IBC and 9 non-IBC samples. It used the expression values of hsa-let-7a, hsa-miR-582-5p, hsa-miR-591 and hsa-mir-16-2-3p as follows. Normalized log-scaled expression values of these miRNAs were combined in a linear combination with weights of -1.73, 1.36, -0.57, and -0.56, respectively. Then the value of this linear combination was compared with a threshold of 0.49, namely the higher values attributed a sample to IBC class and the lower values attributed a sample to non-IBC class. This means that the sample classification performed by this classifier is based on the value of the linear combination:


where *Expr* is a normalized log-scaled expression of the corresponding miRNA for the sample, and positive values of *L* attribute a sample to the IBC class while negative values of *L* attribute a sample to a non-IBC class. In case of the training set this classifier successfully classified 14 samples and 2 IBC samples were misclassified as belonging to non-IBC class resulting in the accuracy of 87.5%. For the testing set comprising 6 IBC and 8 non-IBC samples only one sample was misclassified (a non-IBC sample was attributed to IBC class), and hence the resulting accuracy was 92.9%. The fact that miRNAs from the identified predictive set are not differentially expressed is due to the used algorithm which is aimed at the maximization of a cumulative informative power of a miRNA set irrespectively of the individual informative power of selected miRNAs.

**Figure 2:**
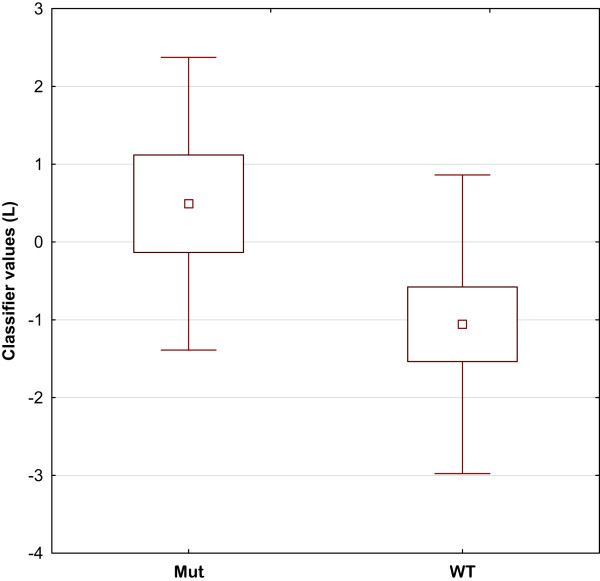
**Association of a predictive set of 4 miRNAs with the TP53 mutational status.** Classifier values *L* for TP53-mutated (Mut) and TP53 wild-type (WT) samples. Smaller bar shows the estimate of the mean value, larger bar shows a 95% confidence interval for the mean value. Vertical line shows mean value ± standard deviation.

We hypothesized that the identified set of 4 miRNAs could be associated with clinico-pathological characteristics of breast cancer patients in general. To test this hypothesis we used published miRNA dataset of 101 breast cancer patient collection with GEO accession number GSE19783 [33]. The analysis revealed significant association of miRNA set with the TP53 mutational status characterized by the p-value of 1.7 × 10^-4^ (Figure 2). The dataset included 64 wild-type TP53 samples and 37 samples with mutated TP53.

The tumor suppressor gene TP53 encodes a transcription factor which possesses multiple functions. It has been reported to often have missense mutations in many cancers compromising its suppressor function. Although current experimental data on the acquisition of oncogenic activities by the mutant forms of this protein are too heterogenous to directly conclude about its impact on tumor development and outcome, TP53 is considered to be an important prognostic marker [73]. In combination with other parameters, e.g., expression profile of selected genes, TP53 mutational status can provide the information on the overall survival and response to treatment for breast cancer patients [74].

**Figure 3:**
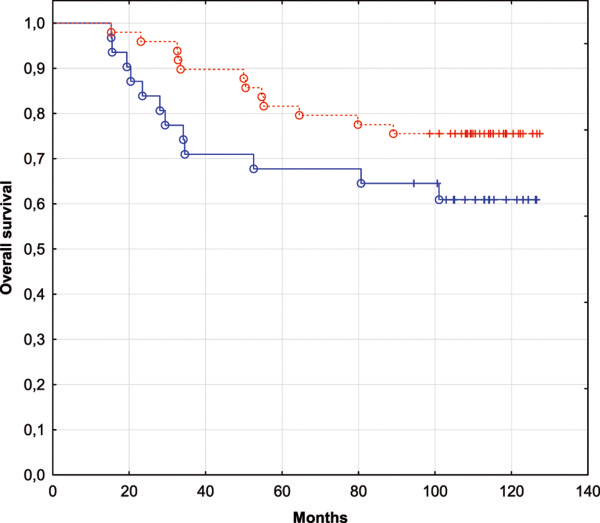
**Kaplan-Meier survival curves for the patients from GSE19783 dataset classified using the IBC-specific predictive set of 4 miRNAs.** The blue curve corresponds to the patients closer to the IBC class (*L* > 0). The red curve corresponds to the patients closer to non-IBC class (*L* < 0).

Figure 3 demonstrates overall survival of the patients from the dataset GSE19783 classified using the same IBC-specific set of 4 miRNAs. The patients with the expression pattern characteristic for IBC have poor prognosis (blue curve) whereas the non-IBC-like patients have better survival (red curve). Although the Cox F-test p-value is only 7.3% here indicating moderate statistical significance, the result is consistent with the clinical value of TP53 status and points out to the functional relevance of IBC-specific miRNA expression pattern.

## Conclusions

We have characterized the complete miRNome of inflammatory breast cancer and revealed differentially expressed miRNAs which reliably classify the patients to IBC and non-IBC groups. We found that the mRNAs and pathways likely regulated by these miRNAs are highly relevant to cancer progression. Also we identified a minimal IBC-related predictive set of 4 miRNAs associated with the TP53 mutational status and survival for breast cancer patients. The described miRNAs should be investigated in future as potential biomarkers and targets for therapy.

## Electronic supplementary material

Additional file 1: **miRNA expression data of the patients.** (XLSX 573 KB)

## Abbreviations

miRNA: microRNA
mRNA: Messenger RNA
IBC: Inflammatory breast cancer.
